# Development of SSR molecular markers and genetic diversity analysis of *Clematis acerifolia* from Taihang Mountains

**DOI:** 10.1371/journal.pone.0285754

**Published:** 2023-05-19

**Authors:** Zhengnan Zhao, Hongwei Zhang, Pingxi Wang, Yuan Yang, Hongyan Sun, Jinyu Li, Xiao Chen, Jun Li, Naizhe Ji, Hui Feng, Shiwei Zhao

**Affiliations:** 1 Beijing Key Laboratory of Greening Plants Breeding, Beijing Academy of Forestry and Landscape Architecture, Beijing, China; 2 Institute of Crop Science, Chinese Academy of Agricultural Sciences, Beijing, China; 3 Henan Institute of Science and Technology, College of Life Science and Technology, Xinxiang, Henan, China; 4 College of Agronomy and Biotechnology, China Agricultural University, Beijing, China; KGUT: Graduate University of Advanced Technology, ISLAMIC REPUBLIC OF IRAN

## Abstract

Investigating the genetic diversity and population structure is important in conserving narrowly distributed plants. In this study, 90 *Clematis acerifolia* (*C*. *acerifolia*) plants belonging to nine populations were collected from the Taihang Mountains in Beijing, Hebei, and Henan. Twenty-nine simple sequence repeats (SSR) markers developed based on RAD-seq data were used to analyze the genetic diversity and population structure of *C*. *acerifolia*. The mean PIC value for all markers was 0.2910, indicating all SSR markers showed a moderate degree of polymorphism. The expected heterozygosity of the whole populations was 0.3483, indicating the genetic diversity of both *C*. *acerifolia* var. *elobata* and *C*. *acerifolia* were low. The expected heterozygosity of *C*. *acerifolia* var. *elobata* (He = 0.2800) was higher than that of *C*. *acerifolia* (He = 0.2614). Genetic structure analysis and principal coordinate analysis demonstrated that *C*. *acerifolia* and *C*. *acerifolia* var. *elobata* showed great genetic differences. Molecular variance analysis (AMOVA) demonstrated that within-population genetic variation (68.31%) was the main contributor to the variation of the *C*. *acerifolia* populations. Conclusively, *C*. *acerifolia* var. *elobata* had higher genetic diversity than *C*. *acerifolia*, and there are significant genetic differences between *C*. *acerifolia* and *C*. *acerifolia* var. *elobata*, and small genetic variations within the *C*. *acerifolia* populations. Our results provide a scientific and rational basis for the conservation of *C*. *acerifolia* and provide a reference for the conservation of other cliff plants.

## Introduction

**Fig 1:**
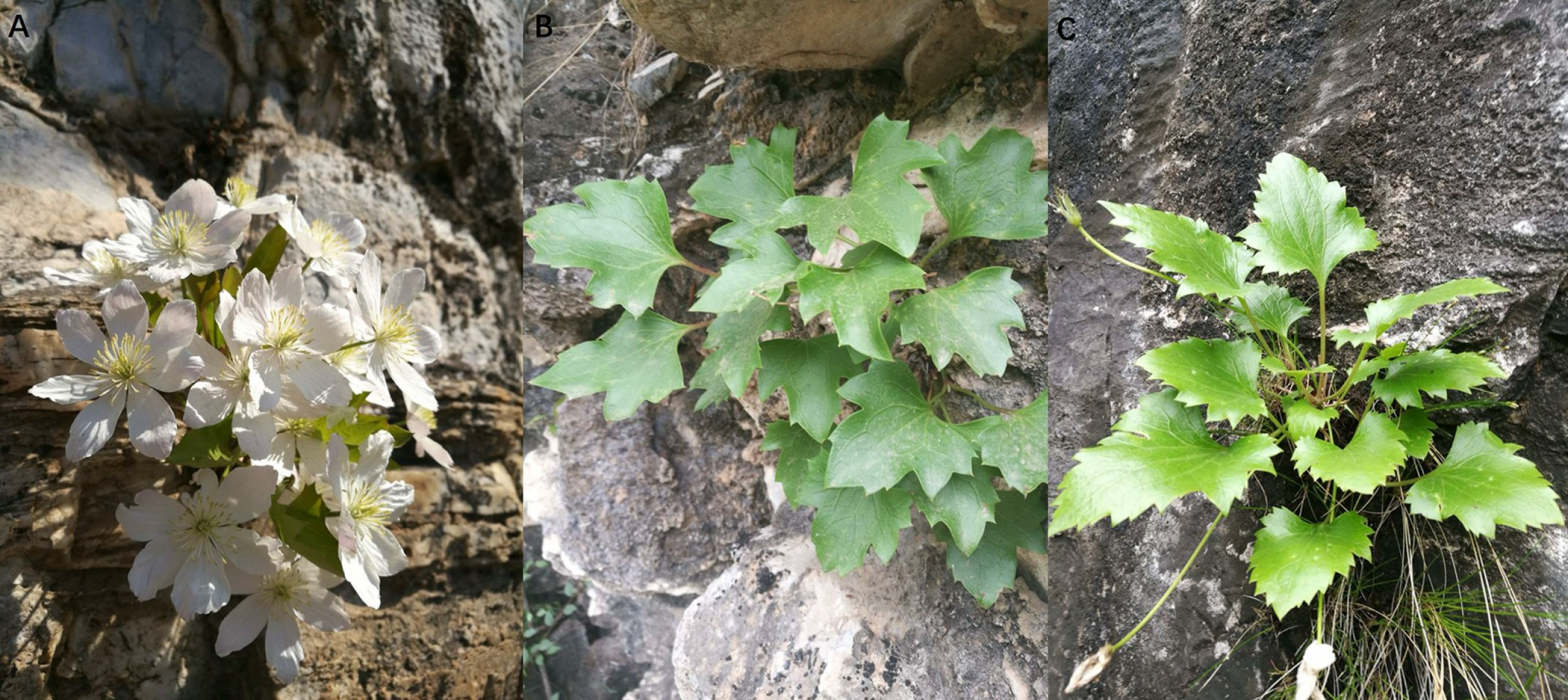
Growth stations of *C*. *acerifolia* and *C*. *acerifolia* var. *elobata* in the Taihang Mountains. A, *C*. *acerifolia* in flowering [image captured in 2019 in Fangshan, Beijing]; B, *C*. *acerifolia*, mainly located in Beijing area, with deeper leaf lobes [image captured in 2019 in Fangshan, Beijing]; and C, *C*. *acerifolia* var. *elobata* distributed in Henan area, with shorter plants and shallow leaf lobes [image captured in 2019 in Jiaozuo, Henan].

*Clematis acerifolia* Maxim. (*C*. *acerifolia*) belongs to the genus *Clematis* L. and the family Ranunculaceae, and is a diploid shrub with white or pink petals [1,2]. It flowers from April to May, and is distributed in a narrow distribution range along the Taihang mountains, mainly inhabiting (**Fig 1**) in the Mentougou and Fangshan districts of Beijing, with sporadic distributions in Henan [3] and Hebei provinces [4]. *C*. *acerifolia* var. *elobata* is a varietas of *C*. *acerifolia*, and is mainly distributed in the Taihang mountains in Henan province. *C*. *acerifolia* is one of the rare and precious plant species that grow on the cliffs of Taihang mountains. Their vertical or nearly vertical growth habit limits water, nutrient, and soil availability, leading to the situation that such plants are usually narrowly distributed, fragmented, and susceptible to environmental influence [5–7]. These cliff-dwelling plant species are strongly tolerant to poor environmental conditions and evolve gradually with long lifespans as well as slow individual growth rates [8]. As a rare and special species in the Taihang mountains, *C*. *acerifolia* has been listed as a second-class protected plant species in China in 2021. Research on the genetic diversity of *C*. *acerifolia* would provide suggestions on the conservation of this species, maintenance of ecological diversity, and development of new varieties for garden landscaping.

Genetic diversity is a key factor for the survival of a species [9], playing an important role in environmental adaptation and long-term survival [10]. High levels of genetic diversity enhance the evolutionary potential, while species with low-level genetic diversity show poor resistance to environmental change [11]. Genetic drift and inbreeding [12] cause plants with narrow distributions to have low genetic diversity, leading to reduced adaptability, reproductive capacity, and disease resistance [13]. Several factors affect the genetic diversity of plants [14], including their effective population size [15], sex ratio [16], mating and breeding systems [17], population life history [18], and gene flow [19]. External factors such as weather [20] and human activity [21] can also affect the evolution of such plants.

Genetic diversity analysis providing suggestions on species conservation and management [22]. As an effective tool for genetic diversity analysis, molecular markers can be used to evaluate the genetic variation and diversity of plants [23,24], and have been used in the genetic study of *Clematis* L. For example, randomly amplified polymorphic DNA (RAPD) and single nucleotide polymorphisms (SNPs) were used for identifying the hybrid origin of progenies derived by crossing *Clematis tubulosa* with *Clematis brevicaudata* [25]. Inter-simple sequence repeat (ISSR), sequence characterized amplified regions (SCAR), chloroplast DNA markers, and internal transcribed spacer (ITS) markers were developed to analyze the genetic diversity and the evolutionary relationship of *Clematis* L. [26–28]. Simple sequence repeat (SSR) marker is a convenient tool in plant genetic and evolutionary analysis due to advantages such as co-dominance, high polymorphism, and high stability [24,29]. SSR markers were used for investigating the genetic relationship among 43 individuals of 11 *Clematis* species [30], and performing the genetic investigation of *Clematis finetiana*, *Clematis chinensis*, and *Clematis heracleifolia* [31–33]. Restriction site-associated DNA sequencing (RAD-seq) is an inexpensive method for developing SSR markers, and have been used in genetic studies of several species [34–36].

Several studies have investigated the genetic diversity of *C*. *acerifolia*. Allozyme marker analysis showed that *C*. *acerifolia* had low genetic diversity in Beijing and Hebei, and revealed that heterozygosity was not significantly associated with adaptation [37,38]. DNA marker analysis revealed the taxonomic position of *C*. *acerifolia* var. *elobata* in the genus *Clematis* L., and found that *C*. *acerifolia* was relatively isolated within the genus *Clematis* L. [2,39]. Considering that the sampling areas were limited and the sample sizes are small in previous studies, it was necessary to collect large numbers of samples from wide areas, and used nuclear genome-based molecular markers to study the genetic classification of *C*. *acerifolia*.

The aim of this study was to (1) develop SSR markers that can assess the genetic diversity and genetic structure of *C*. *acerifolia* and *C*. *acerifolia* var. *elobata* sampled from different locations along the Taihang Mountains, (2) use SSR markers to distinguish *C*. *acerifolia* from *C*. *acerifolia* var. *elobata*, and (3) propose effective conservation measures for *C*. *acerifolia* based on the results of genetic diversity analysis.

## Materials and methods

### Plant materials and DNA extraction

*C*.*acerifolia* only grows in the crevices of cliffs in the Taihang Mountains, showing an extremely narrow and fragmented distribution. Its core distributions are in the south-western part of Beijing and a small part of Hebei (close to Beijing). The *C*. *acerifolia* var. *elobata* is mainly distributed in the south of Taihang Mountains in Jiaozuo, Henan, and was chosen as the southernmost representative population. Nine *C*. *acerifolia* populations, each including ten individuals, were obtained in the study (S1 Table). Populations 1 and 2 were sampled from Zhuozhou, Hebei province; populations 3, 4, 6, 7, 8, and 9 were sampled from Beijing, and population 5 (*C*. *acerifolia* var. *elobata*) was sampled from Jiaozuo, Henan. In order to avoid taking samples from the same crevice, individual samples were taken from distances of at least 3 meters. Samples were collected in 2019, and *C*. *acerifolia* was recognized as a protected plant in 2021. Therefore, there was no need to get permission from any owners to take the samples. Specific information, including location, region, numbering, longitude, latitude, and altitude, is presented in S1 Table. Genomic DNA was extracted using cetyltrimethylammonium bromide (CTAB), and its quality was verified using 1% electrophoresis and diluted to 25 ng·μL^-1^.

### SSR marker development

We prepared the library for RAD sequencing according to a previous study [40]. Briefly, genomic DNA was digested with MseI restriction endonuclease, and the DNA fragments were ligated to P1 adaptor. After that, the ligated sequences were subjected to equal mixing, random breaking, and sequence selection (300-500bp in length). The selected sequences were ligated to P2 adaptor for PCR amplification. The prepared libraries were sequenced on the Illumina Hiseq platform for pair end 150bp sequencing.

Sequencing data preparation was performed according to the method used in a previous study [41]. Briefly, FASTQ software was used to control the quality of raw sequencing data and remove low-quality data. The steps of quality control included: removing adapter sequences; cutting off bases with sequencing quality values < 20 or identified as N at the 5’ end; cutting off bases with sequencing quality values < 3 or identified as N at the 3’ end; cutting off the bases with an average mass value < 20 in a 4-base window; removing reads with N up to 10%; trimming off more than 40% of Reads with base mass less than 15; discarding reads less than 30 bp in length after adapter removal and mass trimming. The number of clean reads, total bases, GC content and Q30 ratio were counted. A quality threshold of Q30 value ≥ 80% was used. The obtained data were publicly available on NCBI (PRJNA849339). The clean reads were assembled with SOAPdenovo2 software [42]. The top 20% scaffolds with longest sequencing data were used for scanning SSR markers. SSRHunter1.3 software [43] was used to search for tandem repeats in each splice sequence. The maximum and minimum numbers of repeating nucleotides were set as 6 and 2, respectively, and the number of repeats was set to be larger than 4. SSR markers were designed with Primer3 (https://bioinfo.ut.ee/primer3-0.4.0/) based on a length of PCR amplifying product ≤ 200 bp. Primer synthesis was performed by the Shanghai Biotechnology Company.

### PCR analysis

Each 10-μL PCR reaction contained 5 μLof Taq Master Mix (Vazyme, Nanjing, China), 1 μL of DNA solution, 0.3 μL of reverser primer, 0.3 μL forward primer, and 3.4 μLof ddH_2_O. PCR was conducted at 94°C for 5 min for pre-denaturation, followed by 30 cycles at 94°C for 30 s, with annealing at 58–62°C for 30 s, elongation at 72°C for 30 s, and finally an extension at 72°C for 7 min. Polyacrylamide gel electrophoresis was performed as stated in a previous study [44] to display the PCR product.

### Analysis of genetic diversity and population structure

We firstly analyze the genetic diversity using the 29 SSR markers. The polymorphic information content (PIC) of each marker was computed with PowerMarkerV3.25 [45], whereas other parameters such as the number of observed alleles (Na), the number of effective alleles (Ne), Shannon’s diversity index (I), observed heterozygosity (Ho), expected heterozygosity (He), and percentage of polymorphic loci (PPL) were computed using POPGENE Version 1.32 [46]. After that, fixation index (*F*_IX_) was calculated as (He-Ho)/He. *F*-statistics [47] including *F*_IS_, *F*_IT_, and *F*_ST_ were calculated using POPGENE Version 1.32. At the same time, we counted the numbers of alleles, polymorphic alleles, common alleles and rare alleles of SSR primers.

We also analyzed the population structure of the samples. Nei’s genetic distance [48] and the genetic identity between populations were calculated using POPGENE Version 1.32. Pairwise population *F*_ST_ and gene flow (Nm) calculation, and principal coordinate analysis (PCoA) were performed using GenALEx6.5 [49]. Molecular variance analysis (AMOVA) was carried out using Arlequin 3.5.2.2 [50]. The genetic identity of 90 individuals were calculated by using NTSYS-pc2.11 [51,52].

## Results

### SSR primers development and characterization

After removing low-quality sequences, ambiguous barcodes and orphan paired-end reads, 161.49 GB of sequences containing 1914 million clean reads were obtained. The average Q30 ratio was 92.9%, ranging from 91.7% to 93.2%, indicating high-quality clean data. The average guanine-cytosine (GC) contents were 39.1% (S2 Table). The length of assembled sequence was 40.83 Mb, including 61653 scaffolds (≥ 500 bp). The longest scaffold was 2.9Mb. The top 20% scaffold were used for designing SSR markers. We obtained 1600 SSR primers and perform polymorphism screening using polyacrylamide gel electrophoresis (S1 Fig).

Two samples from each location (Beijing, Hebei, and Henan) were used for screening the polymorphisms of the 1600 SSR primers. To screen reliable SSR markers, we required that at least one of the six samples had a different band from the other five samples. We selected 29 pairs of markers in the end. Among the 29 SSR markers, the number of markers that had 4, 5, 6, 7, 10, and 13 repeats of tandem sequences were 21, 2, 3, 1, 1, and 1 respectively, and the number of markers that contain 2, 3, and 5 nucleotides in their tandem repeats were 25, 3, and 1, respectively. “AT” was the most common tandem repeat, followed by “TA”, “TC”, “GA”, “AG”, “CA”, “CT”, and “TG” (Table 1). The lengths of PCR products ranged from 127 bp to 198 bp (Table 1), and 29 SSR markers had a total of 92 bands in the nine populations. Four, one, four, seven, and 13 SSRs have 6, 5, 4, 3, and 2 bands, respectively. The number of alleles were 2, 3, and 4, and the number of polymorphic alleles were 1, 2, 3, and 4 (S3 Table).

**Table 1:** The names, sequences, repeat elements, number of repeats, and product lengths of the 29 pairs of simple sequence repeats (SSRs) primers.

SSRs	Forward sequence	Reverse sequence	Repeat motif	Repeat number	Length (bp)
CA63	aaagcagcatggaagcagat	ttgtcttgagtggggtcca	CA	5	182
CA64	gaaccacgccttcaaaactt	ttgcctagagacatgcgttc	TC	4	151
CA75	tctgtgtggaattcgtggtt	agcccgtttgtttcatgatt	TTA	6	193
CA85	atgggggctaggttttgagt	tttcggcttagtttgggttc	AT	4	164
CA122	tcaaaccccaacttgtcaga	tcttggacctctccaagtagat	TA	4	177
CA142	ctcgggagttactgggtcaa	cctaaacccaaacccaaacc	TTG	4	189
CA143	ctcgggagttactgggtcaa	cctaaacccaaacccaaacc	GA	4	189
CA213	ccaaatcatctgcctggaac	gacccccgggttatcacta	TC	4	185
CA255	ggattctcataaggcctactacca	cgatgtctgctgttgaaagg	AT	4	161
CA305	attccaaagttgcgctccta	gcctttcaccttcttctcca	GA	4	166
CA315	aaatgcgtgggcatgtaagt	gtgtgagattccccgtcaac	GGTCC	4	172
CA356	ttctacccttaaaccttcaccaa	tggaagaattgatctcattgga	TA	4	150
CA425	ggttatagatgcatgtgccaat	taggtccaccatgcaggtta	TG	4	172
CA454	ttcttctccacgcattgttg	tcggtcgtgaaggaataagg	CAC	4	157
CA487	gcccaaacagaaacgaaaaa	tgccattattgtttgtgaagc	AT	5	127
CA506	ccgccaacaactccttttt	ggatctcaactccacattgc	GA	4	183
CA551	ttgtagcctatgggcaatgat	aaggggaatcaatcctcacc	AT	6	182
CA554	cccttcgaattcattgcttt	gtaagggcacgtgagaagga	CT	4	155
CA620	tcaaattcccacacctttgag	gggcttggtttaaatgggtaa	AG	4	175
CA635	caaggtcgcaccgatttaac	ggattttcactcaatttttgcat	AG	4	181
CA720	caccatgatcgttgagagga	aacgacacccttttgagtgg	AG	7	177
CA782	gccagttgcctgatcacaag	cctgtttctctgtctccttgg	TC	4	157
CA830	tgcagaacgtgcaattccta	gtgagtccccgaattcattg	AT	6	152
CA997	atgggcctgttggttatgag	acatccaggaggcaaaacat	TA	4	164
CA1091	cttcggatgttcttcagatgc	cacgagcacagaacagaacc	TC	4	168
CA1152	caggaattgtcgaacaaaagg	tgggaattctccaatacatgc	TA	4	162
CA1324	caatttcggaaacatcgaatc	ttcgctcaagactcccaagt	CT	10	164
CA1544	gtgggcgatggagatagaga	tcaccaaaacgcatcagaac	AT	4	198
CA1547	gaagtttacacgcacgctca	tgccaaggaaagagaatcca	CA	13	150

PIC is an important indicator of marker quality and varied from 0.0994 to 0.4888, with a mean value of 0.2910. The PIC values of 12 SSRs were in the range of 0–0.25, and those of 17 SSRs were in the range of 0.25–0.50 (Table 2). F-statistic analysis showed that the mean value of inbreeding coefficient (*F*_*IS*_) in each population was less than 0 (-0.3442) and that the mean value of inbreeding coefficient (*F*_*IT*_) in the whole population was greater than 0 (0.0851), indicating that these loci were mostly heterozygous in each population, and homozygous across the whole population. The *F*_*ST*_ variation ranged from 0.064 to 1, with a mean value of 0.3194, indicating a greater degree of genetic diversity across populations.

**Table 2:** Statistics of 29 SSR markers in nine populations.

SSRs	PIC	*F*_IS_	*F*_IT_	*F*_ST_
**CA63**	0.1400	0.2000	0.7818	0.7273
**CA64**	0.3719	-0.0089	0.2350	0.2418
**CA75**	0.2919	-0.1858	0.4754	0.5576
**CA85**	0.2225	-0.2950	-0.1765	0.0915
**CA122**	0.3207	-0.5823	-0.3846	0.1249
**CA142**	0.4780	-0.6605	-0.3243	0.2025
**CA143**	0.1780	-	1.0000	1.0000
**CA213**	0.3114	-0.7916	-0.3740	0.2331
**CA255**	0.0994	-1.0000	-0.0588	0.4706
**CA305**	0.3719	-1.0000	-0.8000	0.1000
**CA315**	0.4888	-0.0412	0.4910	0.5111
**CA356**	0.0994	-1.0000	-0.0588	0.4706
**CA425**	0.1638	-0.1111	0.8765	0.8889
**CA454**	0.4035	-0.0794	0.2052	0.2636
**CA487**	0.1564	-0.1765	0.8051	0.8344
**CA506**	0.3749	-0.8052	-0.6897	0.0640
**CA551**	0.1780	-	1.0000	1.0000
**CA554**	0.3747	0.7963	0.8888	0.4538
**CA620**	0.0994	-1.0000	-0.0588	0.4760
**CA635**	0.0994	-1.0000	-0.0588	0.4706
**CA720**	0.4625	0.2126	0.3818	0.2149
**CA782**	0.4737	0.1291	0.3939	0.3041
**CA830**	0.1780	-	1.0000	1.0000
**CA997**	0.3012	-0.4716	-0.3235	0.1006
**CA1091**	0.4360	-0.7668	-0.5594	0.1174
**CA1152**	0.3146	-0.0076	0.0746	0.0816
**CA1324**	0.4302	0.0933	0.2399	0.1617
**CA1544**	0.1780	-	1.0000	1.0000
**CA1547**	0.4327	-0.6234	-0.511	0.0693
**Mean**	0.2910	-0.3442	0.0851	0.3194

Notes: PIC is polymorphism information content, which is related to the number and frequency of alleles; *F*_IS_ and *F*_IT_ indicate the probability that one pair of alleles is homozygous in individual population and the whole population, respectively; *F*_ST_ is an indicator of population differences caused by gene inheritance.

### Genetic diversity in *C*. *acerifolia* within populations

Seven parameters were used to measure the genetic diversity: number of observed alleles, number of effective alleles, observed heterozygosity, expected heterozygosity, PPL, Shannon’s diversity index, and fixation index. The results demonstrated (Table 3) that the number of observed alleles of the nine populations varied from 1.5517 to 1.7586 with a mean value of 1.6207, while the number of effective alleles varied from 1.3485 to 1.5176 with a mean value of 1.4299. The distribution of the numbers of observed and effective alleles in different populations was not uniform. Observed heterozygosity ranged from 0.3862 to 0.2621, and expected heterozygosity ranged from 0.2087 to 0.28, showing that observed heterozygosity was higher than expected heterozygosity. *F*_IX_ was less than 0, indicating that there were more heterozygous loci in these populations. PPL ranged from 51.72–65.52%, with a mean value of 58.62%, and Shannon’s diversity index varied from 0.2972 to 0.4011, indicating little variation in the genetic diversity of these populations.

**Table 3:** Genetic diversity parameters of nine *Clematis acerifolia* (*C*. *acerifolia)* populations at the population level and species level.

Population	Na	Ne	I	Ho	He	*F*_IX_	PPL(%)
**1**	1.6207	1.4675	0.3612	0.3207	0.2624	-0.2222	58.62
**2**	1.6207	1.4275	0.3501	0.3103	0.2505	-0.2387	58.62
**3**	1.5862	1.4405	0.3530	0.3448	0.2566	-0.3437	58.62
**4**	1.6207	1.4582	0.3661	0.3138	0.2655	-0.1819	62.07
**5**	1.7586	1.5176	0.4011	0.3862	0.2800	-0.3792	65.52
**6**	1.5862	1.3529	0.2972	0.2621	0.2087	-0.2559	55.17
**7**	1.5517	1.4280	0.3340	0.3034	0.2441	-0.2429	51.72
**8**	1.5862	1.3485	0.3099	0.3000	0.2172	-0.3812	58.62
**9**	1.6552	1.4286	0.3492	0.3103	0.2481	-0.2507	58.62
**Population level**	1.6207	1.4299	0.3469	0.3168	0.2481	-0.2774	58.62
**Species level**	2.4483	1.6310	0.5682	0.3169	0.3483	-	-

Notes: Population level is the mean of the statistic calculated for each population; species level is the statistic obtained with 29 markers across the whole population. Na, the average number of observed alleles; Ne, the average number of effective alleles; I, Shannon′s diversity index; Ho, observed heterozygosity; He, expected heterozygosity; *F*_IX_ = (He-Ho)/He. PPL, band polymorphism; Na and Ne are indicators of the uniformity of gene distribution; Ho, He, and F_IX_ are indicators of the homozygous and heterozygous status of the population; I and PPL are indicators of population diversity.

Comparison of the genetic diversity indices among different populations indicated that these genetic parameters were highest in the *C*. *acerifolia* var. *elobata* population, suggesting that the genetic diversity of *C*. *acerifolia* var. *elobata*. is higher than that of *C*. *acerifolia*. of the *C*. *acerifolia* populations in Beijing and Hebei, populations 1 (Sibeiyu village, Hebei) and 4 (Wuheer Tunnel, Fangshan, Beijing) had higher expected heterozygosity, Shannon’s index, and the number of effective alleles, suggesting higher genetic diversity for these populations. The value for Shannon’s diversity index, expected heterozygosity, and PPL of population 2 and population 3 were similar, and both had comparable levels of genetic diversity, which were lower than those of populations 1 and 4. The lowest number of observed alleles and PPL were found in population 7 (Jingxi Ancient Road), and the observed heterozygosity, expected heterozygosity, and Shannon’s diversity index were low in populations 6 and 8, implying low genetic diversity in populations 6, 7, and 8. The genetic diversity in population 7 was higher than that of populations 6 and 8. Population 9, which is located in Xinghuang Village, had the highest number of observed alleles, suggesting that a higher number of genotypes may have been retained in this population.

Genetic parameters of *C*. *acerifolia* populations from different regions were analyzed at both the species and the population level (Table 4). Except for the number of observed alleles at the species level, the genetic parameters of *C*. *acerifolia* were lower than those of *C*. *acerifolia* var. *elobata*., with similar values in in Beijing and Hebei populations. Generally, genetic diversity was higher at the species level than at the population level.

**Table 4:** Genetic diversity parameters of the *C*. *acerifolia* populations distributed in different regions (Beijing, Hebei, and Henan) at the species level and population level.

Level	Region	Na	Ne	I	Ho	He
**Species level**	Beijing	1.6897	1.4552	0.3776	0.3057	0.2575
	Hebei	1.6897	1.4805	0.3720	0.3155	0.2619
	Henan	1.7586	1.5176	0.4011	0.3862	0.2800
	Beijing+Hebei	1.7586	1.4661	0.3835	0.3082	0.2614
**Population level**	Beijing	1.5977	1.4094	0.3349	0.3057	0.2400
	Hebei	1.6207	1.4475	0.3556	0.3155	0.2564
	Henan	1.7586	1.5176	0.4011	0.3862	0.2800
	Beijing+Hebei	1.6034	1.4189	0.3400	0.3082	0.2441

Notes: In this table, genetic diversity-related parameters were calculated in four regions. Beijing and Hebei (near Beijing) represent the main distribution areas. Na, the average number of observed alleles; Ne, the average number of effective alleles; I, Shannon′s diversity index; Ho, observed heterozygosity; He, expected heterozygosity. Na and Ne are indicators of the uniformity of gene distribution; Ho and He are indicators of the homozygous and heterozygous status of the population, respectively; I is an indicator of population diversity.

### Genetic diversity among populations

Genetic diversity among the populations was measured based on genetic distance, genetic identity, pairwise population *F*_ST_, and gene flow. The genetic distances (S4 Table) varied from 0.0148 to 0.9603 among the nine populations. In particular, the genetic distance between the *C*. *acerifolia* and *C*. *acerifolia* var. *elobata*. was greater than 0.8, and the genetic identity was less than 0.44. The genetic distances between the *C*. *acerifolia* populations in Beijing and Hebei ranged from 0.0148 to 0.0734, all of which were less than 0.1, and the genetic identities were greater than 0.92, with pairwise population *F*_ST_ (S5 Table) varying between 0.023 and 0.469, indicating that *C*. *acerifolia* was genetically distant from *C*. *acerifolia* var. *elobata*, and that the groups comprising *C*. *acerifolia* in Beijing and Hebei were genetically close to each other. The *F*_ST_ between the *C*. *acerifolia* and *C*. *acerifolia* var. *elobata* populations was greater than 0.25, and the gene flow was less than 1, demonstrating a large genetic diversity. The maximum *F*_ST_ value of 0.108 for *C*. *acerifolia* populations and a gene flow greater than 1 indicated that frequent gene exchange has occurred in the *C*. *acerifolia* populations in Beijing and Hebei. These results indicated significant genetic differences between the populations in Henan, Hebei, and Beijing. The genetic differences between the populations sampled from Beijing and Hebei were minimal, with populations that were moderately genetically differentiated or largely undifferentiated. These results suggest that the *C*. *acerifolia* var. *elobata* in Henan has significantly independent genetic diversity compared to the Hebei and Beijing *C*. *acerifolia* populations.

### Genetic structure of populations

**Fig 2:**
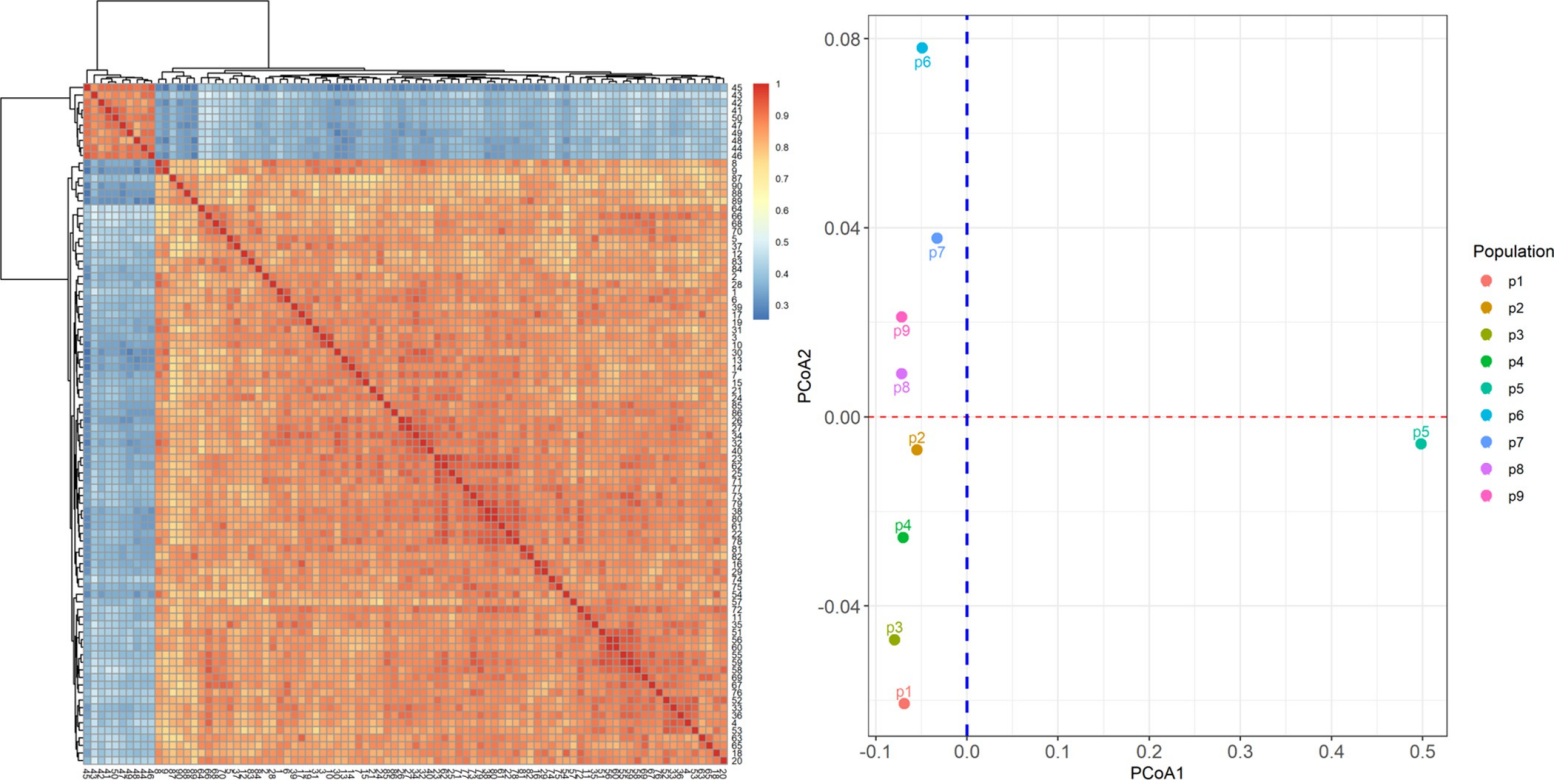
The relationship of 90 *C*. *acerifolia* individuals. Notes: (A) showed clustering based on the genetic identity matrix; (B) shows that the material can be divided into 2 principal components based on principal coordinate analysis.

The genetic structure of the populations was analyzed by PCoA, cluster analysis, and AMOVA. Cluster analysis was performed using the samples from 90 individuals (Fig 2A). The 90 individuals were divided into two classes with a genetic identity of 0.38. The first category comprised the *C*. *acerifolia* var. *elobata* samples, which were all from Henan and were numbered from 41 to 50, while the second category contained 80 individuals from Hebei and Beijing. The results of PCoA were used to divide the nine populations into two principal components (Fig 2B), the first of which can explain 84.56% of the phenotypic variation and the second 5.2% of the variation. The *C*. *acerifolia* var. *elobata* distributed in Henan was distinguishable from the *C*. *acerifolia* population, which was split into two groups. The AMOVA results (Table 5) revealed that the nine populations distributed in the three locations had 31.69% inter-population variation and 68.31% intra-population variation with an *F*_ST_ of 0.3169, indicating significant genetic variation among the populations. The *F*_ST_ value for *C*. *acerifolia* was 0.078, and the genetic variation was mainly within-population variation, accounting for 92.19% of the total variation. These results indicate significant genetic differences between *C*. *acerifolia* and *C*. *acerifolia* var. *elobata*, with less genetic variation within the *C*. *acerifolia* populations in Beijing and Hebei.

**Table 5:** Molecular variance analysis (AMOVA).

Population	Source of variation	df	Sum of squares	Variance of components	Percentage of variance
**All the populations**	Across populations	8	292.917	1.652	31.69
	Within populations	171	609.050	3.562	68.31
	Total	179	901.967	5.214	
**Populations in Beijing and Hebei**	Across populations	7	66.312	0.298	7.81
	Within populations	152	534.200	3.514	92.19
	Total	159	600.513	3.812	

Notes: AMOVA divided the sources of variations into within and across population variations; df, degrees of freedom.

## Discussion

### Advantages of SSR molecular markers for analyzing the genetic diversity of *C*. *acerifolia*

SSR marker can estimate genomic variation in highly variable regions by amplifying fragment differences in higher eukaryotic genomes [53]. SSR repeats commonly consist of one to seven base pairs, with advantages including co-dominant, universal, highly informative polymorphism, and abundant information [54], rendering SSR a favorable tool for measuring plant genetic diversity. PIC is a key indicator that can be obtained from SSR markers, reflecting the number of alleles and the relative abundance of gene frequencies in a population [23,24]. PIC values can be divided into three levels: high polymorphism (>0.5), moderate polymorphism (0.25–0.5), and low polymorphism (<0.25) [55]. The PIC values for the markers in this study ranged from 0.094 to 0.4888, with a mean value of 0.2910, indicating moderate polymorphism.

In contrast to molecular markers developed from plastid genome of *C*. *acerifolia* [39], we developed SSR markers using RAD-seq data from the nuclear genome to perform genetic diversity of *C*. *acerifolia*. The *F*_ST_ values between the populations from the three provinces greater than 0.25 suggesting a significant divergence between *C*. *acerofolia* and *C*. *acerofolia* var. *elobata*. The results of genetic distance, genetic identity, gene flow analysis, PCoA, and NJ clustering were consistent with the above results and showed that the markers could be used to distinguish *C*. *acerofolia* var. *elobata* from *C*. *acerofolia*. The developed primers can therefore be used to assess the genetic diversity of *C*. *acerofolia* populations in different geographical locations, providing a scientific basis for analyzing their population status.

### Population genetic diversity

Compared with widely distributed plants, endemic species that are narrowly distributed in specialized and limited environments have relatively small populations and are susceptible to fragmentation due to external disturbances. Therefore, it is challenging to conserve the narrowly distributed endemic plants. Compared to other narrowly distributed plants in China, *C*. *acerofolia* and *C*. *acerofolia* var. *elobata*. have lower than expected heterozygosity (Tables 3 and 4), whereas cliff cypress (*Thuja sutchuenensis*, He = 0.23–0.53, mean = 0.395) [13], Guizhou golden camellia (*Camellia huana*, He = 0.466) [56], and *Rosa odorata* var. *gigantea* (He = 0.569) [57] all showing higher expected heterozygosity. In comparison to narrowly distributed species (He = 0.56), endemic species (He = 0.42), and widely distributed plants (He = 0.62), the expected heterozygosity of the population in this study is low [58]. *Taihangia rupestris* var. *ciliata* (Na = 10.2, Ho = 0.55, He = 0.787) [59] and *Opisthopappus taihangensis* (Na = 3.9, Ho = 0.653, He = 0.472) [60], which are also distributed around Taihang mountains, have higher number of observed alleles, observed heterozygosity, and expected heterozygosity than those obtained for the nine populations in this study (Na = 2.4483, Ho = 0.3169, He = 0.3483). The above results demonstrate the low genetic diversity of both *C*. *acerifolia* var. *elabata* and *C*. *acerifolia*.

### Genetic structure is influenced by specific habitat

The living environment and reproduction of plants are closely related to the genetic structure of a population. As a habitat for many rare plants, cliffs are a special habitat with fragmented distribution and high heterogeneity [61]. Cliff plants are subject to high environmental selection pressure, with every cliff crevice providing a microenvironment with different conditions. Cliff plants propagate through methods appropriate to their lifestyles during their long-term evolution. Similar to cliff plants such as *T*. *rupestris* and *Oxyria sinensis* [61], *C*. *acerifolia* populations with strongly branched rhizomes can grow along stone crevices [62]. Small numbers of seedlings are also occasionally observed in the wild [37].

Genetic variation can be divided into inter- and intra-population differences. The variation within the *C*. *acerifolia* population observed in this study (92.19%) was higher than that across populations (7.81%). This intra-genetic variation is derived from selection due to the cliff environment and is complemented by new sporadic seedlings. First, the environmental selection of particular ecotypes contributes to genetic diversity. During the long evolutionary process, plants adapt to and are selected by environments, especially species in heterogeneous microenvironments, where competition can be highly aggressive [63]. Plants with high adaptability to environments will survive, contributing to high genetic diversity within the population. Second, new seedlings replenish stock during the long evolutionary period, with survival strategies such as long lifespans, slow growth rate, and slow seedling replenishment used by many cliff plants [8]. Although the emergence of new *C*. *acerifolia* seedlings is infrequent, the occasional and irregular complementation with seedlings can also provide a source of genetic variation and prevent genetic drift, as found by Araki [64], Zhao [65], Watkinson and Powell [66].

Genetic variation among populations is largely dependent on gene flow, with seeds and pollen acting as vehicles. Studies have shown that *C*. *acerifolia* seeds spread mainly by gravity, while spider webs on the cliffs retain seeds in the same area [37]. The seeds have a tailed hair structure that leads to them becoming entangled during maturation, increasing the gravity of the "seed mass" and preserving the seeds close to the cliff habitat to some degree [37]. The tailed hairs on the seeds help the plant to disperse following maturation, causing them to germinate in the cliff crevices. Thus, *C*. *acerifolia* seeds are more likely to spread within their original or adjacent populations, with a smaller probability of dispersal between Beijing and Henan, which are at a greater distance. The biological mechanism by which pollination takes place in *C*. *acerifolia* remains unclear. Flower-visiting organisms such as bees and beetles observed in the wild may be potentially pollinators for this plant. The large genetic variation between *C*. *acerifolia* and *C*. *acerifolia* var. *elobata* suggests that seed or pollen flow transmission is small, and the low genetic variation among the populations of *C*. *acerifolia* indicates that seed or pollen transmission is high.

### Comparison with published genetic diversity of *C*. *acerifolia*

A related study in 2005 used allozyme to analyze the genetic diversity of *C*. *acerifolia* in Beijing and Hebei. The results of the present study using SSR molecular markers demonstrated low genetic diversity for *C*. *acerifolia*. The degree of genetic diversity obtained in the two studies differs. According to the *F*_ST_ values, the results of the former study showed large genetic differences between the *C*. *acerifolia* populations in Beijing and Hebei, while the present study demonstrated only a moderate degree of genetic variation. Such differences may be caused by temporal changes in the genetic diversity of the populations, the use of different molecular markers, and variation in the samples from various sampling sites. Inbreeding decline is a problem commonly faced by cliff plants and is closely related to the production of homozygous offsprings. Contrary to the previous assessments of inbreeding in *C*. *acerifolia* populations, the inbreeding coefficient (*F*_IS_) was less than 0 and the Ho is greater than He in this study, indicating a more heterozygous state in these loci. The possible reason for this situation is the harsh environment of cliff plants, with less interspecific competition and more intraspecific competition [67]. Related studies have shown that heterozygous plants in species experiencing strong competition may become dominant and that the genetic diversity of populations is maintained through the continuous evolution of heterozygous plants [63]. In contrast, highly homozygous plants are less resistant to environmental changes and are prone to mortality. In this study, 29 SSR primers were used to evaluate the overall inbreeding level of the plants, and revealed that this species showed more heterozygoty than homozygoty.

### Conservation measures for *C*. *acerifolia*

Previous studies have shown that the life span of cliff plants is generally long, probably ranging from 45 to 324 years [68]. The individual *C*. *acerifolia* plants may also live for long periods. Habitat changes, intra-population competition, and random plant mortality may be responsible for the reduced genetic diversity of this population.

The following conservation measures are recommended based on the genetic diversity assessment and cluster analysis of *C*. *acerfolia* in this study. First, attention should be paid to protecting habitats. *C*. *acerifolia* has a unique habitat and can only grow in the cliff environments of the Taihang Mountains, thus requiring protection via actions such as lowering human interference and minimizing the reduction of population genetic diversity due to habitat destruction. Second, in situ conservation is important to protect existing plants. *C*. *acerifolia* populations are renewing and evloving slowly, and the individuals live for long periods; therefore, in situ conservation can maintain or delay decreases in genetic diversity. At the same time, the conservation of wild seedlings is particularly critical because of their contribution to genetic diversity. Thirdly, various methods are required to increase the number of plants. A seedling establishment can be maintained by artificially raising seedlings, which can then be returned to their native habitat. It may also be possible to place seeds directly into the crevices of cliffs. The survey also showed that sheep climb the cliff and eat the plants; therefore, grazing should be prohibited in areas where the plant is concentrated.

Lastly, the results of this study indicate that the *C*. *acerifolia* and *C*. *acerifolia* var. *elobata* are genetically different and that each is an important component of the total genetic diversity. The Beijing and Hebei groups have lower genetic diversity and are thus more vulnerable, and the conservation of these groups should be prioritized. Genetic diversity should be maximized as much as possible during conservation. Populations 4 (Wuheer Tunnel, Beijing) and 1 (Sibeiyu Village, Hebei) had high genetic diversity, and populations 6 (Nanshiyang Grand Canyon, Beijing), 7 (Jingxi Ancient Road, Beijing), and 8 (Xiayun Hill, Beijing) had low genetic diversity. Therefore, for populations in Nanshiyang Grand Canyon, Jingxi Ancient Road, and Xiayun Hill, measures should be taken to protect the habitats and propagate seedlings for re-entry into the ecosystem. Population 9 in Xinghuang Village has the highest number of observed alleles, and conserving this species in this location can significantly protect the diversity of all genotypes. At the same time, introducing populations with distinct genetic characteristics from the south of Taihang mountains (such as Henan) to Beijing is an advisable way to improve the genetic diversity of *C*.*acerifolia*. Artificial crossing of Henan and Beijing populations will accelerate the improvement of genetic diversity in Beijing.

## Conclusion

In this study, we used SSR markers to genotype plants belonging to *C*. *acerifolia* and *C*. *acerifolia* var. *elobata* respectively at the DNA level, and evaluated the genetic diversity of *C*. *acerifolia* populations sampled from different locations. Our results showed that the nine populations had low genetic diversity and that *C*. *acerifolia* var. *elobata* had higher genetic diversity than *C*. *acerifolia*. The molecular markers developed from the nuclear genome enabled a more comprehensive evaluation of genetic differences.

## Supporting information

S1 Fig The electrophoresis gel of CA315 and CA213. The numbers under the gel represent the genotype numbering.
(TIF)

S1 Table Basic information of the nine *C*. *acerifolia* populations. (DOCX)

S2 Table Statistics of RAD-seq data. (DOCX)

S3 Table The numbers of alleles, polymorphic alleles, common alleles and rare alleles of SSR primers. (DOCX)

S4 Table Genetic identity (up-right) and nei′s genetic distance (bottom-left) of the nine *C*. *acerifolia* populations. (DOCX)

S5 Table Pairwise population *F*_*ST*_ (bottom-left) and gene flow (Nm, up-right) among different *C*. *acerifolia* populations. (DOCX)
