# Supplementary figures and images for: Development of SSR molecular markers and genetic diversity analysis of Clematis acerifolia from Taihang Mountains
Source: PLoS One. 2023 May 19;18(5):e0285754. doi: 10.1371/journal.pone.0285754 (PMC10198494; doi:10.1371/journal.pone.0285754)

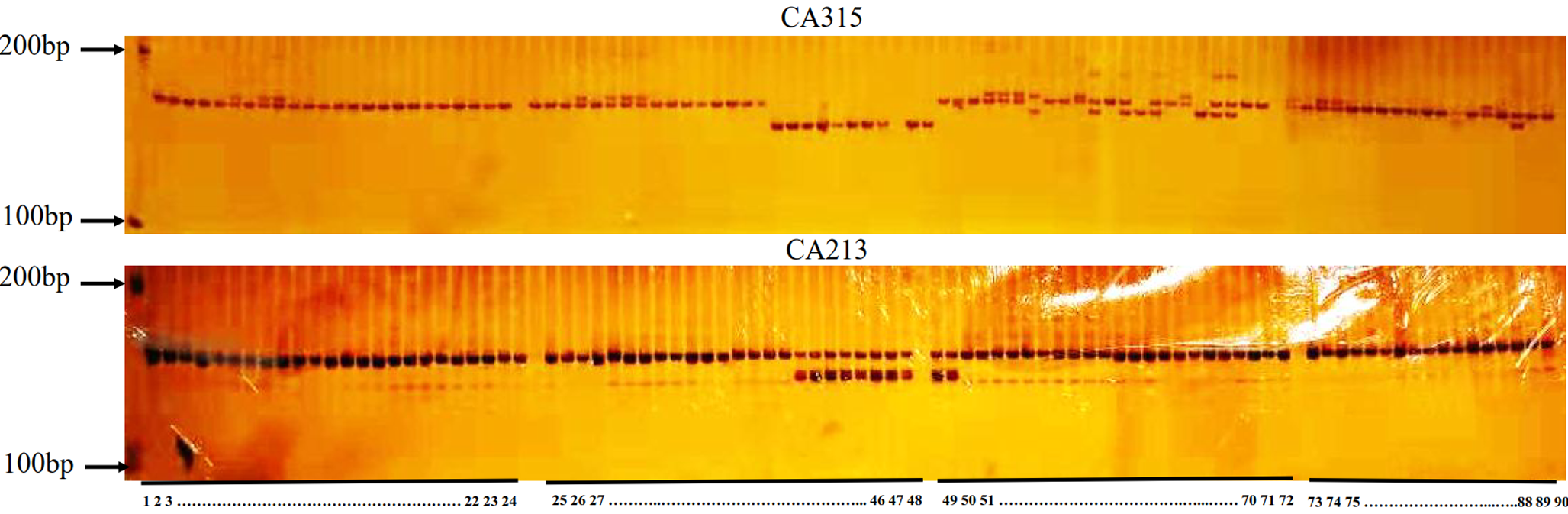

Supplement: S1 Fig — The numbers under the gel represent the genotype numbering. (TIF) [file pone.0285754.s001.tif]
