# Supplementary material for: Development of SSR molecular markers and genetic diversity analysis of Clematis acerifolia from Taihang Mountains
Source: PLoS One. 2023 May 19;18(5):e0285754. doi: 10.1371/journal.pone.0285754 (PMC10198494; doi:10.1371/journal.pone.0285754)
Supplement: S1 Table — (DOCX) [file pone.0285754.s002.docx]

**S1 Table. Basic information of the nine** ***Clematis acerifolia* （*C. acerifolia* ）populations (each population containing 10 individuals), including distribution location, distribution area, number of samples, code number, longitude, latitude, and altitude of the sampling sites.**

| **Species** | **Population** | **Locations** | **Regions** | **Population size** | **Numbering** | **Latitude** | **Longitude** | **Altitude (meter)** |
| --- | --- | --- | --- | --- | --- | --- | --- | --- |
| ***C. acerifolia*** | 1 | Sibeiyu Village | Hebei | 10 | 1-10 | N 39°39' | E 115°28' | 279.3 |
|  | 2 | Gougezhuang Village | Hebei | 10 | 11-20 | N 39°41' | E 115°25' | 185.7 |
|  | 3 | Fangshan Sandu | Beijing | 10 | 21-30 | N 39°37' | E 115°39' | 129.0 |
|  | 4 | Wuheer Tunnel | Beijing | 10 | 31-40 | N 39°39' | E 115°36' | 199.8 |
| ***C. acerifolia* var. *elobata*** | 5 | Yuntai mountains | Henan | 10 | 41-50 | N 35°26' | E 113°22' | 524.5 |
| ***C. acerifolia*** | 6 | Nanshiyang Grand Canyon | Beijing | 10 | 51-60 | N 40°03' | E 115°50' | 315.5 |
|  | 7 | Jingxi Ancient Road | Beijing | 10 | 61-70 | N 39°59' | E 116°02' | 147.8 |
|  | 8 | Xiayun Hill | Beijing | 10 | 71-80 | N 39°48' | E 115°47' | 257.9 |
|  | 9 | Xinghuang Village | Beijing | 10 | 81-90 | N 39°43' | E 115°47' | 469.3 |
