# Supplementary material for: Development of SSR molecular markers and genetic diversity analysis of Clematis acerifolia from Taihang Mountains
Source: PLoS One. 2023 May 19;18(5):e0285754. doi: 10.1371/journal.pone.0285754 (PMC10198494; doi:10.1371/journal.pone.0285754)
Supplement: S2 Table — (DOCX) [file pone.0285754.s003.docx]

**Table S2 Statistics of RAD-seq data.**

| **Sample** | **Clean reads** | **Clean base** | **GC(%)** | **Q30(%)** |
| --- | --- | --- | --- | --- |
| **1** | 5276634 | 1557169679 | 38.79 | 92.89 |
| **2** | 5310220 | 1567121866 | 38.88 | 93.07 |
| **3** | 4940608 | 1457790330 | 38.85 | 93.14 |
| **4** | 5589932 | 1649903767 | 38.94 | 93.01 |
| **5** | 5374343 | 1586217598 | 39.11 | 93.02 |
| **6** | 5782381 | 1718348048 | 38.85 | 93.18 |
| **7** | 4930417 | 1465050213 | 38.94 | 92.96 |
| **8** | 6280297 | 1866467159 | 39.01 | 93.19 |
| **9** | 6276700 | 1858811415 | 38.89 | 93.2 |
| **10** | 5219801 | 1545963074 | 38.98 | 93.18 |
| **11** | 5517120 | 1622710722 | 39.4 | 93 |
| **12** | 6475049 | 1904324481 | 39.67 | 92.78 |
| **13** | 5945720 | 1748329183 | 39.79 | 91.98 |
| **14** | 6561686 | 1929589578 | 40.27 | 92.87 |
| **15** | 5717425 | 1675452711 | 38.9 | 92.82 |
| **16** | 4971153 | 1457125398 | 39.18 | 92.86 |
| **17** | 4836826 | 1417379455 | 39.04 | 93.02 |
| **18** | 5077518 | 1488179751 | 38.93 | 92.9 |
| **19** | 5151898 | 1509779157 | 39.08 | 92.19 |
| **20** | 6615290 | 1939222789 | 40.08 | 92.84 |
| **21** | 5443774 | 1596030073 | 39.41 | 92.92 |
| **22** | 4696992 | 1377179327 | 39.25 | 92.9 |
| **23** | 5904321 | 1742867726 | 39.36 | 92.77 |
| **24** | 5489571 | 1620518131 | 39.33 | 92.96 |
| **25** | 5577697 | 1646360967 | 39.3 | 93.02 |
| **26** | 5487615 | 1619990513 | 39.32 | 92.95 |
| **27** | 6011879 | 1774465946 | 40.54 | 92.82 |
| **28** | 5433360 | 1614777631 | 39.09 | 93.14 |
| **29** | 5295425 | 1573761619 | 39.02 | 92.95 |
| **30** | 5989184 | 1780089680 | 39.1 | 93.2 |
| **31** | 5118223 | 1516005709 | 38.97 | 93.14 |
| **32** | 4744497 | 1405367160 | 39.07 | 93.16 |
| **33** | 4580623 | 1347408912 | 38.99 | 93.02 |
| **34** | 5206314 | 1531423971 | 39.1 | 92.78 |
| **35** | 5022615 | 1477242059 | 39.17 | 92.01 |
| **36** | 5275463 | 1551971127 | 39.07 | 93.05 |
| **37** | 5572345 | 1633167631 | 39.01 | 92.89 |
| **38** | 4486965 | 1315429464 | 39.13 | 92.88 |
| **39** | 4409820 | 1292742173 | 38.92 | 93.05 |
| **40** | 4994977 | 1464248369 | 39.35 | 92.86 |
| **41** | 5750140 | 1685435531 | 38.78 | 92.24 |
| **42** | 5910376 | 1732643084 | 38.79 | 92.99 |
| **43** | 5987669 | 1755037057 | 38.7 | 92.98 |
| **44** | 5956879 | 1746167304 | 38.86 | 93.01 |
| **45** | 6712851 | 1981255243 | 38.69 | 92.83 |
| **46** | 6068476 | 1790954397 | 38.73 | 92.98 |
| **47** | 5852743 | 1727181093 | 38.93 | 93.11 |
| **48** | 5859299 | 1729395196 | 38.73 | 93.08 |
| **49** | 6980862 | 2060497612 | 38.68 | 93.11 |
| **50** | 7262040 | 2158174846 | 38.95 | 93.2 |
| **51** | 7096794 | 2109352986 | 38.88 | 92.96 |
| **52** | 6922130 | 2057251915 | 38.97 | 93.22 |
| **53** | 7200943 | 2132782621 | 39.01 | 93.19 |
| **54** | 6941509 | 2056054130 | 38.93 | 93.22 |
| **55** | 5564759 | 1636800691 | 39.24 | 93.03 |
| **56** | 6512030 | 1915460677 | 38.99 | 92.81 |
| **57** | 6448397 | 1896713072 | 39.1 | 92.01 |
| **58** | 5616442 | 1652157549 | 39.35 | 93.04 |
| **59** | 5925124 | 1736601637 | 39.18 | 92.88 |
| **60** | 7338699 | 2151589387 | 39.1 | 92.9 |
| **61** | 4899957 | 1436210417 | 39.25 | 92.74 |
| **62** | 5103559 | 1496133159 | 39.04 | 92.58 |
| **63** | 4807993 | 1409386481 | 39.11 | 91.87 |
| **64** | 6630576 | 1944135979 | 39.54 | 92.57 |
| **65** | 5926879 | 1737748604 | 39.15 | 92.65 |
| **66** | 5815131 | 1704964875 | 39.87 | 92.53 |
| **67** | 6229876 | 1838961295 | 39.14 | 92.5 |
| **68** | 5542291 | 1636013492 | 39.05 | 92.69 |
| **69** | 5092702 | 1502954281 | 39.1 | 92.77 |
| **70** | 5451562 | 1609324330 | 38.99 | 92.71 |
| **71** | 5569606 | 1644044825 | 39.24 | 92.68 |
| **72** | 5629986 | 1673256421 | 39.01 | 92.82 |
| **73** | 6035797 | 1793914139 | 39.14 | 92.65 |
| **74** | 6236708 | 1853720516 | 39.23 | 92.83 |
| **75** | 5653126 | 1674164794 | 39.29 | 92.77 |
| **76** | 5936093 | 1758295683 | 39 | 92.87 |
| **77** | 5054955 | 1486950012 | 39.16 | 92.66 |
| **78** | 6946329 | 2042760497 | 39.09 | 92.35 |
| **79** | 5596211 | 1646101709 | 39.07 | 91.7 |
| **80** | 5668393 | 1667529580 | 39.09 | 92.73 |
| **81** | 6602841 | 1934840559 | 39.58 | 92.92 |
| **82** | 6665062 | 1953437991 | 39.09 | 92.99 |
| **83** | 5375341 | 1575237392 | 39.27 | 93.12 |
| **84** | 5844861 | 1712627113 | 39.17 | 93.02 |
| **85** | 5705616 | 1671973592 | 39.21 | 92.25 |
| **86** | 5938574 | 1740752916 | 39.08 | 93.02 |
| **87** | 5690840 | 1667735580 | 39.07 | 93.1 |
| **88** | 5569013 | 1631874120 | 38.96 | 93.14 |
| **89** | 6405739 | 1890065028 | 39.05 | 92.95 |
| **90** | 5739155 | 1693503585 | 39.08 | 93.09 |

Notes:

Sample: sample code.

Clean Reads: high-quality reads number.

Clean Base: The total number of bases of high-quality sequencing data remaining after filtering of the raw data.

Q30: The percentage of bases in Clean Data that have a mass value greater than or equal to 30.

GC: GC content in clean data
