# Supplementary material for: Development of SSR molecular markers and genetic diversity analysis of Clematis acerifolia from Taihang Mountains
Source: PLoS One. 2023 May 19;18(5):e0285754. doi: 10.1371/journal.pone.0285754 (PMC10198494; doi:10.1371/journal.pone.0285754)
Supplement: S3 Table — (DOCX) [file pone.0285754.s004.docx]

**S3 Table. The numbers of alleles, polymorphic alleles, common alleles and rare alleles of SSR primers.**

| **SSRs** | **Number of alleles** | **number of polymorphic alleles** | **number of common alleles** | **number of rare alleles** |
| --- | --- | --- | --- | --- |
| **CA63** | 2 | 2 | 0 | 0 |
| **CA64** | 2 | 2 | 0 | 0 |
| **CA75** | 4 | 4 | 0 | 0 |
| **CA85** | 2 | 2 | 0 | 0 |
| **CA122** | 2 | 1 | 1 | 0 |
| **CA142** | 3 | 3 | 0 | 0 |
| **CA143** | 2 | 2 | 0 | 0 |
| **CA213** | 2 | 1 | 1 | 0 |
| **CA255** | 2 | 1 | 1 | 0 |
| **CA305** | 2 | 1 | 1 | 0 |
| **CA315** | 4 | 4 | 0 | 0 |
| **CA356** | 2 | 1 | 1 | 0 |
| **CA425** | 2 | 2 | 0 | 0 |
| **CA454** | 3 | 3 | 0 | 0 |
| **CA487** | 2 | 2 | 0 | 0 |
| **CA506** | 2 | 2 | 0 | 0 |
| **CA551** | 2 | 2 | 0 | 0 |
| **CA554** | 2 | 2 | 0 | 0 |
| **CA620** | 2 | 1 | 1 | 0 |
| **CA635** | 2 | 1 | 1 | 0 |
| **CA720** | 4 | 4 | 0 | 2 |
| **CA782** | 4 | 4 | 0 | 0 |
| **CA830** | 2 | 2 | 0 | 0 |
| **CA997** | 2 | 2 | 0 | 0 |
| **CA1091** | 3 | 2 | 1 | 0 |
| **CA1152** | 2 | 2 | 0 | 0 |
| **CA1324** | 3 | 3 | 0 | 0 |
| **CA1544** | 2 | 2 | 0 | 0 |
| **CA1547** | 3 | 3 | 0 | 1 |

Note: Number of rare alleles indicate alleles with a gene frequency of less than 5%.
