# Supplementary material for: Development of SSR molecular markers and genetic diversity analysis of Clematis acerifolia from Taihang Mountains
Source: PLoS One. 2023 May 19;18(5):e0285754. doi: 10.1371/journal.pone.0285754 (PMC10198494; doi:10.1371/journal.pone.0285754)
Supplement: S4 Table — (DOCX) [file pone.0285754.s005.docx]

**S4 Table. Genetic identity (up-right) and genetic distance (nei′s 1972) (bottom-left) of the** **nine *Clematis acerifolia* (*C. acerifolia* ) populations.**

| **Population** | **1** | **2** | **3** | **4** | **5** | **6** | **7** | **8** | **9** |
| --- | --- | --- | --- | --- | --- | --- | --- | --- | --- |
| **1** |  | 0.9690 | 0.9812 | 0.9853 | 0.3959 | 0.9292 | 0.9455 | 0.9544 | 0.9533 |
| **2** | 0.0315 |  | 0.9624 | 0.9796 | 0.4128 | 0.9629 | 0.9564 | 0.9581 | 0.9408 |
| **3** | 0.0189 | 0.0383 |  | 0.9795 | 0.3828 | 0.9324 | 0.9508 | 0.9781 | 0.9501 |
| **4** | 0.0148 | 0.0206 | 0.0207 |  | 0.3975 | 0.9577 | 0.9598 | 0.9734 | 0.9595 |
| **5** | 0.9266 | 0.8849 | 0.9603 | 0.9226 |  | 0.4155 | 0.4384 | 0.3916 | 0.3887 |
| **6** | 0.0734 | 0.0378 | 0.0699 | 0.0432 | 0.8783 |  | 0.9604 | 0.9618 | 0.9453 |
| **7** | 0.0561 | 0.0446 | 0.0504 | 0.0410 | 0.8247 | 0.0405 |  | 0.9578 | 0.9547 |
| **8** | 0.0467 | 0.0428 | 0.0221 | 0.0270 | 0.9375 | 0.0390 | 0.0431 |  | 0.9458 |
| **9** | 0.0479 | 0.0610 | 0.0512 | 0.0414 | 0.9450 | 0.0562 | 0.0464 | 0.0557 |  |
