# Supplementary material for: Development of SSR molecular markers and genetic diversity analysis of Clematis acerifolia from Taihang Mountains
Source: PLoS One. 2023 May 19;18(5):e0285754. doi: 10.1371/journal.pone.0285754 (PMC10198494; doi:10.1371/journal.pone.0285754)
Supplement: S5 Table — (DOCX) [file pone.0285754.s006.docx]

**S5 Table. Pairwise population *F*_ST_ (*F*_ST_, bottom-left) and gene flow (Nm, up-right) among different *Clematis acerifolia* (*C. acerifolia*) populations.**

| **Population** | **1** | **2** | **3** | **4** | **5** | **6** | **7** | **8** | **9** |
| --- | --- | --- | --- | --- | --- | --- | --- | --- | --- |
| **1** | 0 | 5.432 | 6.694 | 10.619 | 0.296 | 2.064 | 2.691 | 3.373 | 3.174 |
| **2** | 0.044 | 0 | 4.380 | 7.563 | 0.304 | 3.481 | 3.175 | 3.596 | 2.528 |
| **3** | 0.036 | 0.054 | 0 | 6.160 | 0.288 | 2.154 | 2.955 | 6.000 | 2.955 |
| **4** | 0.023 | 0.032 | 0.039 | 0 | 0.295 | 2.875 | 3.175 | 4.652 | 3.987 |
| **5** | 0.458 | 0.451 | 0.465 | 0.459 | 0 | 0.308 | 0.311 | 0.283 | 0.291 |
| **6** | 0.108 | 0.067 | 0.104 | 0.080 | 0.448 | 0 | 3.039 | 3.917 | 2.623 |
| **7** | 0.085 | 0.073 | 0.078 | 0.073 | 0.446 | 0.076 | 0 | 3.321 | 2.726 |
| **8** | 0.069 | 0.065 | 0.040 | 0.051 | 0.469 | 0.060 | 0.070 | 0 | 2.726 |
| **9** | 0.073 | 0.090 | 0.078 | 0.059 | 0.462 | 0.087 | 0.084 | 0.084 | 0 |

Notes: The *F*_ST_ was greater than 0.25, and the gene flow was less than 1, demonstrating large genetic differences. The *F*_ST_ value less than 0.15 and the gene flow greater than 1 indicate that frequent gene exchange has occurred.
